# The impact of the zero-waste city pilot policy on the synergistic reduction of CO₂ and air pollutant emissions: evidence from China

**DOI:** 10.3389/fpubh.2026.1853126

**Published:** 2026-06-19

**Authors:** Bingnan Guo, Yuren Qian, Feng Hu, Hao Hu, Ji Lin

**Affiliations:** 1 School of Humanities and Social Sciences, Jiangsu University of Science and Technology, Zhenjiang, China; 2 Institute of International Business & Economics Innovation and Governance, Shanghai University of International Business and Economics, Shanghai, China; 3 School of Economics, Shanghai University, Shanghai, China; 4 School of Economics and Management, Wenzhou University of Technology, Wenzhou, China

**Keywords:** circular economy, environmental exposure, public health, synergistic control of CO₂ and air pollutant emissions, zero-waste city pilot

## Abstract

Against the backdrop of China’s carbon peaking and carbon neutrality agenda and intensified pollution control efforts, advancing the synergistic control of CO₂ and air pollutant emissions has become an important pathway toward green transformation and the reduction of environmental health risks. Using panel data for 286 prefecture-level cities in China from 2011 to 2022, this study treats the zero-waste city pilot policy as a quasi-natural experiment and applies a double machine learning framework to identify its impact on urban synergistic outcomes in CO₂ and air pollutant emission control. The results show that: (1) the zero-waste city pilot significantly improves the synergistic control of CO₂ and air pollutant emissions, and this finding remains robust across multiple checks; (2) the policy effect operates mainly through source reduction, resource recycling, and high-standard harmless treatment; and (3) the effects are more pronounced in central and western regions, in small- and medium-sized cities, and in non-resource-based cities. Overall, this study provides new evidence that integrated environmental governance can simultaneously promote environmental sustainability and public health improvement.

## 1 Introduction

In recent years, China’s ecological and environmental quality has improved steadily; nevertheless, structural tensions between conventional pollutant emissions and carbon emissions remain pronounced, and the long-standing challenges of high aggregate energy consumption and low resource-use efficiency have yet to be fundamentally alleviated. According to the 2024 Bulletin on the State of China’s Ecological Environment, air-quality standards are still unmet in some regions nationwide, while ecosystem stability and environmental carrying capacity continue to face multiple, compounding pressures (1). To better coordinate economic development with ecological security, the Party Central Committee and the State Council have introduced a series of major strategic initiatives, most notably the carbon peaking and carbon neutrality (“dual-carbon”) goals. Key policy documents—including the 14th Five-Year Plan for Comprehensive Work on Energy Conservation and Emissions Reduction and the Implementation Plan for the Synergistic Control of CO₂ and Air Pollutant Emissions (CCA)—underscore the need to advance carbon mitigation, pollution reduction, ecological enhancement, and economic growth in an integrated manner, and to establish a long-term institutional framework for green and low-carbon development. Accordingly, CCA has become not only a central task of ecological-civilization development but also a strategic underpinning of high-quality growth (2). Since the issuance of the Work Plan for the Pilot Program of “Zero-Waste City” Development in 2018, the Zero-Waste City Pilot (ZWCP) has promoted institutional innovation to advance solid-waste minimization, recycling, and safe disposal, thereby offering a new pathway for coordinating urban-level pollution prevention and carbon mitigation while potentially generating important public health co-benefits. However, existing studies on ZWCP have largely concentrated on its performance in solid-waste governance, while systematic identification of its effects on CCA remains comparatively limited. In response, this study takes ZWCP as the focal policy intervention and employs a double machine learning–based causal inference framework to rigorously assess its policy effect on CCA, with particular attention to its public health relevance in reducing pollution-related environmental risks and supporting healthier urban development.

CCA represents a key pathway to achieving a “triple dividend” of high-quality economic development, ecological-civilization advancement, and public health improvement, and it serves as a central lever for green transformation (3). Accordingly, CCA has become not only a central. Because conventional air pollutants and CO₂ emissions are frequently co-generated—sharing common sources, processes, and underlying mechanisms—coordinated governance can deliver multiple environmental, economic, and public health benefits at lower overall cost by simultaneously reducing harmful emissions and population exposure (4). In recent years, a rapidly expanding literature has examined CCA, generating increasingly rich evidence. In terms of research focus, one strand centers on measurement and identification strategies, commonly applying coupling–coordination models, total factor productivity decomposition frameworks, and bidirectional synergy indices to quantify the interlinked dynamics between pollutant abatement and carbon reduction (5). A second strand investigates the synergistic governance effects of policy instruments. For example, low-carbon city pilots have been shown to enhance the efficiency of coordinated urban governance (6); carbon emissions trading schemes can reduce both CO₂ and air pollutants through market-based mechanisms (7); and green finance reform and innovation pilot zones may induce concurrent declines in pollution and carbon emissions by fostering technological innovation and optimizing capital structures (8). A third strand explores underlying mechanisms and transmission channels, emphasizing pathways such as industrial upgrading, green technological innovation, improvements in energy efficiency, and factor-allocation optimization (9). Overall, existing research has shifted from single-target mitigation toward a synergy-oriented perspective, yet it has largely concentrated on evaluating established policy instruments; empirical identification of CCA under emerging governance models such as ZWCP remains relatively limited.

ZWCP constitutes a significant institutional innovation for advancing solid-waste source reduction, high-efficiency resource circulation, and coordinated pollution governance, and it is therefore pivotal to green and low-carbon transition as well as to the attainment of CCA-related objectives. In recent years, a growing body of research has systematically examined the environmental and economic performance of ZWCP and has produced substantial findings. At the aggregate level, some studies evaluate the policy’s abatement and development effects, showing that ZWCP can significantly enhance urban green total factor productivity and carbon-emission efficiency, while facilitating regional green and low-carbon transition through green innovation and spatial spillovers. Complementary evidence suggests that the policy can generate a “win–win” outcome by simultaneously strengthening pollution control and supporting economic growth. At the micro level, scholars have documented firm behavioral responses, finding that ZWCP promotes corporate green innovation by intensifying environmental regulation and improving financing conditions, with stronger effects observed among state-owned enterprises and in regions with greater innovative capacity (10). In addition, corporate ESG performance appears to improve markedly in response to policy incentives. Related work further employs life-cycle assessment, material flow accounting, and LMDI decomposition to verify—through a carbon-footprint lens—the policy’s mitigation potential and its role in improving resource-use efficiency (11). Overall, existing studies largely concur that ZWCP delivers tangible gains in green innovation, resource recycling, and pollution abatement; nevertheless, systematic identification and mechanism-based analysis of its effects on CCA remain comparatively underdeveloped.

However, while the environmental and economic effects of coordinated pollution and carbon control have received increasing attention, relatively limited research has explicitly examined such policy interventions from the perspective of their broader public health implications. First, in terms of research perspective, much of the literature concentrates on single dimensions—such as emissions abatement, green innovation, or resource circulation—while providing relatively limited systematic identification of ZWCP’s synergistic effects on CCA. Second, methodologically, mainstream empirical work largely relies on conventional causal inference approaches (e.g., difference-in-differences and synthetic control), which may be less capable of precisely identifying policy effects in the presence of high-dimensional covariates; concerns related to model-specification bias and heterogeneous treatment effects have not been fully addressed. Third, regarding mechanism analysis, prior research has documented channels linked to resource-use efficiency, technological innovation, and pollution control, but offers less granular evidence on the internal transmission processes and regionally differentiated patterns through which ZWCP may influence CCA.

To address these gaps, this study makes three incremental contributions. First, from a research perspective, it embeds ZWCP within a CCA governance framework and provides a city-level, systematic assessment of its synergistic effects, thereby extending the evidence base on how urban environmental governance may generate broader public health co-benefits. Second, at the methodological level, it introduces a double machine learning approach within a quasi-natural-experiment setting to identify the causal effect of ZWCP, alleviating biases associated with high-dimensional controls and improving the robustness and interpretability of the estimates. Third, in terms of mechanisms, it examines three channels—source reduction, resource recycling, and high-standard harmless treatment—to elucidate how ZWCP advances CCA, offering new empirical evidence and policy implications for strengthening urban ecological-civilization governance and accelerating green and low-carbon transition.

## 2 Policy background and theoretical analysis

### 2.1 Policy background

Against the backdrop of tightening global resource constraints and escalating environmental pollution, solid-waste governance has increasingly become a critical bottleneck constraining urban sustainable development. Internationally, the “zero-waste” has given rise to a diverse set of governance approaches, offering valuable reference points for China (12). Japan, for example, institutionalized the “reduce, reuse, recycle” (3R) principle through the Basic Act for Establishing a Sound Material-Cycle Society and built a waste-governance system characterized by coordinated engagement among government, enterprises, and the public. The European Zero Waste Alliance proposed a “seven-tier hierarchy” that emphasizes a shift from “waste management” to “resource management,” highlighting the primacy of source reduction (13). Singapore’s Zero Waste Masterplan 2030 has been supported by legislation, economic incentives, and technological innovation, forming a governance paradigm tailored to high-density urban settings (14). Sweden, drawing on extended producer responsibility and energy recovery, has reportedly reduced the landfilling rate of municipal waste to below 1% and achieved a decline in carbon emissions of more than 70% relative to 1990 levels (15). Collectively, these experiences suggest that broad-based societal participation, coherent and system-wide policy design, and a well-functioning circular utilization system constitute core institutional pillars for advancing “zero-waste” development (16).

Building on these international lessons, China has gradually explored an indigenous pathway for ZWCP that aligns with national conditions. Guided by the concept of green development, ZWCP aims to realize full-process governance spanning source reduction–resource recycling–high-standard harmless treatment. In June 2018, the Central Commission for Deepening Overall Reform incorporated the construction of “zero-waste cities” into the key tasks of the national pollution prevention and control campaign. In December of the same year, the General Office of the State Council issued the Work Plan for the Pilot Program of “Zero-Waste City” Development, selecting an “11 + 5” set of pilot cities and regions nationwide—including Shenzhen, Baotou, and Tongchuan—to initiate implementation. Subsequently, the Ministry of Ecology and Environment and other agencies released a series of policy documents (e.g., Opinions on Deepening Municipal Solid Waste Classification and Resource Utilization and the 14th Five-Year Plan Implementation Plan for ZWCP Construction), further clarifying ZWCP’s strategic role in advancing the dual-carbon agenda and ecological-civilization development. Over time, the pilot scope expanded to nearly 100 cities, forming a cross-departmental and cross-regional collaborative governance architecture. The names of ZWCP pilot areas are reported in Table 1.

**Table 1:** ZWCP areas.

Categories	Name
Pilot cities	Baotou, Chongqing, Panjin, Ruijin, Sanya, Shaoxing, Shenzhen, Tongling, Weihai, Xuchang, Xining, Xuzhou
Pilot special zones	Beijing Economic and Technological Development Area, China-Singapore Tianjin Eco-City, Guangze, Xiong’an New Area

### 2.2 Theoretical analysis

ZWCP can be understood as a modern urban governance model guided by the new development philosophy, aimed at fostering greener modes of production and consumption and, ultimately, shaping a circular societal development pattern (17). Its policy logic lies in the combined effects of institutional constraints, technological progress, and behavioral guidance to deliver full-process governance over solid waste—spanning source reduction, resource recycling, and high-standard harmless treatment—and to promote cleaner production and circular utilization within the economic and social system. Relative to conventional end-of-pipe approaches, ZWCP places greater emphasis on systemic, value-chain-wide management. By facilitating closed-loop resource flows, it helps shift urban metabolism from “linear growth” to “circular symbiosis,” thereby providing structural support for achieving CCA (18).

*H1*: ZWCP generates positive effects on CCA.

#### 2.2.1 Source reduction

Source reduction represents the most forward-looking component of the 3R principles in the circular economy (19). Its core objective is to reduce resource inputs and waste generation at the production and consumption stages, thereby shifting governance from ex post remediation toward ex ante prevention. Compared with end-of-pipe treatment, ZWCP—through policy steering and institutional innovation—embeds reduction-oriented principles into lifecycle-based urban management, fundamentally lowering resource consumption and environmental burdens. This mechanism operates through two main pathways. First, a production-side pathway of coordinated emissions reduction: by promoting cleaner production, green manufacturing, and material cycling, ZWCP incentivizes firms to reduce resource intensity via process optimization, input substitution, and energy-efficiency management, thereby decreasing solid waste and conventional pollutant emissions while also curbing energy-related carbon emissions. Second, a consumption-side pathway of coordinated demand reduction: by encouraging green consumption and sustainable lifestyles—such as the use of reusable products, green procurement, and low-carbon mobility—ZWCP reduces municipal waste and single-use product consumption, lowering lifecycle carbon footprints at the consumption stage. Through the bidirectional coupling of production-side and consumption-side adjustments, source reduction simultaneously conserves resource inputs and reduces emissions, yielding a systemic synergistic effect on CCA.

*H2*: ZWCP improves CCA by promoting source reduction.

#### 2.2.2 Resource recycling

Resource recycling constitutes one of the core pathways through which ZWCP advances CCA. Its essence is to establish cross-industry and cross-regional cycles of resources and energy, enabling waste-to-resource conversion and energy regeneration. Building on source reduction, this mechanism further enhances resource efficiency and environmental performance. It emphasizes “turning waste into resources” and “using recycling to enable governance,” and seeks to couple material and energy flows across industrial, agricultural, and residential sectors to form closed-loop circular-economy value chains (20). In the industrial domain, ZWCP facilitates circular transformation of industrial parks and symbiotic utilization of by-products among firms, promoting industrial symbiosis and cascading energy use, reducing primary resource extraction and energy-intensive production processes, and thereby lowering both pollutant and greenhouse-gas emissions. In the residential domain, it promotes the integration of recycling networks with municipal waste-sorting systems, improving the circulation and reprocessing efficiency of recyclables and reducing the environmental burdens associated with landfilling and incineration. In the agricultural domain, it supports the conversion of crop residues into feed, fuel, and fertilizer, mitigating open burning and uncontrolled waste disposal. By extending material lifetimes, improving energy-use efficiency, and reducing disposal demand, resource recycling lowers primary resource consumption and cuts carbon emissions from waste-treatment processes, thus generating synergistic gains for CCA (21).

*H3*: ZWCP improves CCA by promoting resource recycling.

#### 2.2.3 High-standard harmless treatment

High-standard harmless treatment serves as a critical safeguard through which ZWCP advances CCA. The core logic is to coordinate the treatment of heterogeneous solid-waste streams and build an intensive, integrated governance system that raises utilization rates and treatment efficiency while reducing pollutant emissions and carbon emissions. This mechanism emphasizes city-level integration of waste-treatment resources by promoting unified collection, classification, and efficient treatment of municipal solid waste, sludge, construction waste, hazardous waste, and agricultural residues, thereby forming a multi-waste coordinated governance pattern. For instance, bottom ash from waste-to-energy incineration can be managed within an integrated system and routed to construction-waste utilization facilities to produce recycled building materials, enabling further reuse. Technologically, advanced processes—such as aerobic composting, anaerobic digestion, and waste-heat recovery—can improve energy efficiency in treatment stages and reduce harmful emissions (22). Institutionally, incorporating harmless-treatment facilities into urban public infrastructure supports a pluralistic co-governance structure characterized by government leadership, enterprise participation, and social oversight (23). Beyond ensuring environmental safety, high-standard harmless treatment can maximize carbon mitigation through the joint effects of pollution control and energy recovery, thereby strengthening the dual gains embedded in CCA.

*H4*: ZWCP improves CCA by establishing a high-standard harmless treatment system.

## 3 Research design

### 3.1 Model setting

The realization of CCA is typically shaped by multiple, jointly operating factors. Accordingly, when evaluating policy-induced synergistic effects, it is essential to control for potential confounders as comprehensively as possible. However, conventional regression frameworks often perform inadequately in settings with high-dimensional covariates and potentially nonlinear relationships: they are prone to the curse of dimensionality and severe multicollinearity, which can weaken identification and compromise robustness. Double machine learning (DML), building on machine-learning and regularization techniques, addresses these challenges by selecting predictive combinations of relevant controls from a pre-specified high-dimensional set and by accommodating nonlinearity and higher-order interactions through flexible functional approximation. In this way, DML helps overcome key limitations of traditional causal inference approaches in high-dimensional, nonlinear environments. Following Chernozhukov et al. (24), this study specifies a partially linear DML model as follows:


$$Y_{i}=\theta_{0}D_{i}+g(X_{i})+U_{i}$$ (1)

$$D_{i}=m(X_{i})+V_{i}$$ (2)

In these equations, $Y_{i}$ denotes the CCA; $D_{i}$ is a dummy that equals 1 once the city joins the ZWCP programme and 0 otherwise; $X_{i}$ represents a potentially high-dimensional vector of control variables; $g(X_{i})$ and $m(X_{i})$ are unknown, flexible functions; and $U_{i}$ and $V_{i}$ are random disturbance terms. The parameter of interest, $\theta_{0}$ , captures the causal effect of the ZWCP policy on CCA. Furthermore:


$$E(U_{i}|D_{i},X_{i})=0$$ (3)

From Equation 1, 2 , and 3 , the unbiased estimator can be obtained as:


$${\overset{ˆ}{\theta }}_{0}={\left[\frac{1}{N}\sum_{i=1}^{N}{\left(D_{i}-\hat{m}(X_{i})\right)}^{2}\right]}^{-1}\cdot [\frac{1}{N}\sum_{i=1}^{N}(D_{i}-\hat{m}(X_{i}))(Y_{i}-\hat{g}(X_{i}))]$$ (4)

Subtracting $\hat{g}(X_{i})$ from both sides of Equation 1, we have:


$$Y_{i}-\hat{g}(X_{i})=\theta_{0}D_{i}+g(X_{i})-\hat{g}(X_{i})+U_{i}$$ (5)

Substituting Equations 2 and 5 into Equation 4, we find that we can decompose into three parts (see Equations 6–8):


$$\frac{1}{\sqrt{N}}\sum_{i=1}^{N}V_{i}(Y_{i}-\hat{g}(X_{i}))$$ (6)

a. The product of two error terms, specifically:


$$\frac{1}{\sqrt{N}}\sum_{i=1}^{N}\;U_{i}V_{i}\overset{P}{∼}N(0,\sigma_{\mathrm{UV}}^{2})$$ (7)

b. The regularized bias product term, namely:


$$\frac{1}{\sqrt{N}}\sum_{i=1}^{N}(\hat{g}(X_{i})-g(X_{i}))(\hat{m}(X_{i})-m(X_{i}))$$ (8)

Even if the convergence rates of both estimation errors are relatively slow, their product in regularization will converge faster than $N^{-1/4}$ , thus becoming an infinitesimal term as N→∞ .

c. The product of error terms and estimation errors, specifically:


$$\frac{1}{\sqrt{N}}\sum_{i=1}^{N}V_{i}(\hat{g}(X_{i})-g(X_{i})),\frac{1}{\sqrt{N}}\sum_{i=1}^{N}U_{i}(\hat{m}(X_{i})-m(X_{i}))$$ (9)

As shown in Equation 9, as the sample size N increases, this term diverges, implying that the estimation bias does not vanish asymptotically; instead, it can grow with the sample size. The bias arises primarily because the same sample used to estimate $\overset{ˆ}{m}(\cdot )$ contains information that may be correlated with $V_{i}$ , thereby inducing dependence between $V_{i}$ and the nuisance-function estimation error $\overset{ˆ}{g}(X_{i})-g(X_{i})$ . To eliminate this source of bias, double machine learning incorporates cross-fitting: by using sample splitting and out-of-fold predictions when estimating nuisance components, the procedure ensures that the target parameter is estimated in an approximately unbiased and consistent manner.

### 3.2 Variable definition

#### 3.2.1 Dependent variable

The dependent variable in this study is the synergistic control of CO₂ and air pollutant emissions (CCA). We first standardize the three subsystems—pollution, carbon emissions, and economic development—and then employ a coupling coordination degree model to quantify the extent of synergy among them. The selection of these indicators is grounded in the theoretical framework of coordinated environmental governance. Specifically, PM2.5 reflects local air pollution control outcomes, CO₂ emissions capture climate mitigation performance, and economic development represents the constraint and objective of sustainable growth. The integration of these three dimensions allows us to evaluate whether cities can achieve simultaneous improvements in pollution reduction and carbon mitigation without sacrificing economic development, which is the core connotation of coordinated control. This index is intended to reflect city-level cca under the context of the zero-waste city policy framework.

Specifically, we proceed as follows. First, for each subsystem (pollution, carbon emissions, and economic development), we apply range standardization (min–max normalization) to the underlying indicator set to eliminate dimensional and scale effects. Second, based on the coupling coordination framework, we compute the coupling–coordination relationship among the three subsystems. Let the composite indices of the pollution subsystem, the carbon-emissions subsystem, and the economic-development subsystem be denoted by $PM_{2.5}、CO_{2}$ and rgdp , respectively. The overall coordination index, the coupling degree, and the coupling coordination degree are then defined as follows (see Equations 10–12):


$$T=\mathrm{\alpha P}M_{2.5}+\mathrm{\beta C}O_{2}+\text{γrgdp}$$ (10)

$$C=\frac{{\left(PM_{2.5}\times CO_{2}\times \text{rgdp}\right)}^{1/3}}{PM_{2.5}+CO_{2}+\text{rgdp}}$$ (11)

$$\mathrm{CCA}=\sqrt{C\times T}$$ (12)

Here, T denotes the overall coordination index, C represents the coupling degree, and CCA is the final coupling coordination degree used to measure the level of synergy among pollution, carbon emissions, and economic development. We set α=β=γ=1/3 , reflecting the assumption that the three subsystems carry relatively balanced importance weights. The resulting value is taken as the city-level *cca*: larger values indicate stronger coordination among pollution abatement, carbon mitigation, and economic growth.

#### 3.2.2 Core explanatory variable

The key explanatory variable is the zero-waste city pilot policy (ZWCP). Based on the first-batch “11 + 5” list of ZWCP pilot cities and regions released by the State Council in May 2019, we designate 2019 as the policy’s implementation start year. The variable is constructed as follows: for pilot cities, *zwcp* is set to 1 from 2019 onward (inclusive), indicating exposure to the policy, and to 0 in 2018 and earlier years; for non-pilot cities, *zwcp* equals 0 throughout the entire sample period. This coding scheme distinguishes cities before and after policy implementation and separates treated from untreated cities, thereby facilitating identification of the net effect of ZWCP on *cca*.

#### 3.2.3 Control variables

Although the double machine learning framework can substantially alleviate the “curse of dimensionality” associated with rich covariate sets, the formation of *cca* is jointly shaped by multiple factors. To isolate the net effect of *zwcp*, we include a set of additional covariates capturing other potential determinants as control variables. To further improve predictive performance and accommodate potential nonlinear relationships, we additionally incorporate the quadratic terms of these controls. Moreover, we include city fixed effects and year fixed effects to absorb unobserved, time-invariant city characteristics and common macro-level time trends that could otherwise confound the estimates. Definitions of the main variables are summarized in Table 2.

**Table 2:** Variable definition.

Variable	Definition
Synergistic control of CO₂ and air pollutant emissions (*cca*)	The degree of coordination among pollution, CO₂ emissions, and economic development
Zero-waste city pilot policy (*zwcp*)	Equals 1 in the implementation year and thereafter for pilot cities; 0 otherwise
Population density (*lnpd*)	Natural logarithm of population per square kilometer
Internet development level (*lnidl*)	Natural logarithm of the number of Internet users
Science and technology input intensity (*rd*)	Ratio of science and technology expenditure to local fiscal expenditure
Financial development level (*fdl*)	Year-end deposit balance of financial institutions as a share of GDP
Government intervention (*gi*)	Local general budget revenue as a share of GDP
Trade openness (*fo*)	Total value of goods imports and exports as a share of GDP
Educational resources (*edu*)	Number of enrolled college students per 10,000 people
Transport accessibility (*lnta*)	Natural logarithm of highway passenger transport volume

#### 3.2.4 Data description and descriptive statistics

This study uses a panel dataset covering 286 prefecture-level cities in China over the period 2011–2022. The data are drawn primarily from the China City Statistical Yearbook, the China Energy Statistical Yearbook, the China Industry Statistical Yearbook, provincial statistical yearbooks, city-level statistical bulletins on national economic and social development, and the CEIC/China Economic Information Network statistical database. To ensure data availability and the reliability of the empirical analysis, we exclude Tibet as well as Hong Kong, Macao, and Taiwan, together with a small number of cities with substantial missing information. For sporadic missing entries, we apply interpolation to impute values. The final sample comprises 286 cities and 3,432 valid city-year observations. Descriptive statistics for all variables are reported in Table 3.

**Table 3:** Descriptive statistic.

Type	Variable	Obs	Mean	SD	Min	Max
Explained variables	*cca*	3,432	0.485	0.059	0.000	0.875
Core explanatory variable	*zwcp*	3,432	0.122	0.328	0.000	1.000
Control variables	*lnpd*	3,432	5.716	0.914	1.792	7.943
	*lnidl*	3,432	13.469	0.958	9.210	15.978
	*lnrd*	3,432	0.014	0.030	0.000	0.936
	*fdl*	3,432	1.505	0.729	0.371	20.100
	*gi*	3,432	0.076	0.025	0.023	0.239
	*fo*	3,432	0.184	0.308	0.000	3.209
	*edu*	3,432	0.018	0.021	0.000	0.129
	*lnta*	3,432	7.941	1.270	1.609	12.184

## 4 Empirical analysis

### 4.1 Baseline estimation results

We empirically evaluate the effect of ZWCP on CCA using a double machine learning framework. In the baseline specification, we adopt a 1:4 sample-splitting scheme and implement random forests as the learner for nuisance-function estimation. To assess robustness, the model is estimated sequentially by adding (i) the first-order control variables, (ii) their quadratic terms, and (iii) year fixed effects and city fixed effects. Table 4 reports the baseline results. Across specifications, the estimated coefficient on ZWCP is positive and statistically significant at the 1% level, indicating that the policy is associated with a significant improvement in city-level CCA.

**Table 4:** Baseline regression result.

Var	*cca*	*cca*	*cca*	*cca*
	(1)	(2)	(3)	(4)
*zwcp*	0.026^***^	0.025^***^	0.027^***^	0.026^***^
	(0.003)	(0.003)	(0.004)	(0.004)
Control (single term)	Y	Y	Y	Y
Control (quadratic term)	N	Y	Y	Y
City FE	N	N	N	Y
Year FE	N	N	Y	Y
N	3,432	3,432	3,432	3,432

The dependent variable is the synergistic control of CO₂ and air pollutant emissions. Column (1) includes the linear terms of the control variables. Column (2) adds the quadratic terms of the control variables. Column (3) includes year fixed effects. Column (4) includes all control variables and fixed effects. Robust standard errors are reported in parentheses. *p < 0.01, *p < 0.05, *p < 0.1.

### 4.2 Robustness checks

#### 4.2.1 Interactive fixed effects

Cities within the same province may share geographic and institutional characteristics, generating correlated shocks and common trends. To strengthen identification and enhance the credibility of the estimates, we adopt a more stringent specification by augmenting the baseline model with province-by-year interactive fixed effects, while retaining city fixed effects and year fixed effects. This specification captures heterogeneous, time-varying provincial trends and helps absorb province-level unobservables that could systematically bias the estimates. Column (1) of Table 5 shows that, after introducing these high-dimensional fixed effects, the estimated coefficient on ZWCP remains significantly positive, indicating that the policy’s effect on CCA is robust.

**Table 5:** Robustness check I.

Var	Interactive fixed effects	Sample adjustment	1%	5%
	(1)	(2)	(3)	(4)
*zwcp*	0.026^***^	0.025^***^	0.024^***^	0.025^***^
	(0.004)	(0.004)	(0.003)	(0.003)
Control (single term)	Y	Y	Y	Y
Control (quadratic term)	Y	Y	Y	Y
City FE	Y	Y	Y	Y
Year FE	Y	Y	Y	Y
*N*	3,432	3,432	3,432	3,432

The dependent variable is the synergistic control of CO₂ and air pollutant emissions. Column (1) adds interactive fixed effects. Column (2) adjusts the study sample. Columns (3) and (4) apply two-sided winsorization at the 1 and 5% levels for continuous variables, respectively. Robust standard errors are reported in parentheses. *p < 0.01, *p < 0.05, *p < 0.1.

#### 4.2.2 Sample adjustment

China’s municipalities directly under the central government differ markedly from prefecture-level cities in administrative status and institutional arrangements. Pooling them with other cities may therefore compromise comparability and affect inference. To mitigate this concern, we exclude the four municipalities and re-estimate the model using the remaining cities only. As reported in Column (2) of Table 5, the coefficient on ZWCP remains positive and statistically significant at the 1% level, implying that the policy continues to significantly improve city-level CCA. This result further corroborates the baseline findings.

#### 4.2.3 Excluding extreme values

The sample may contain outliers that, if unaddressed, could distort identification of ZWCP’s effect on CCA and reduce estimation accuracy. To limit potential bias from extreme observations, we winsorize the main control variables (excluding the policy indicator) using two-sided thresholds at the 1 and 5% quantiles—replacing values beyond the cutoffs with the corresponding boundary values—and then re-estimate the model. Columns (3) and (4) of Table 5 indicate that, under both winsorization schemes, the estimated coefficient on ZWCP remains positive and significant at the 1% level, underscoring the robustness of the policy effect on CCA.

#### 4.2.4 Elimination of outliers

Outliers in the research sample may bias the estimated carbon-mitigation effect of the ZWCP. To secure both accuracy and robustness, we winsorise every control variable—other than the policy indicator itself—at the 1st/99th and 5th/95th percentiles, respectively, replacing observations that fall outside these bounds with the corresponding cutoff values and then re-estimate the model. As reported in columns (4) and (5) of Table 5, the ZWCP coefficient remains significantly negative under both winsorisation schemes, indicating that the policy indeed promotes a reduction in carbon emissions. This finding further confirms the reliability of the baseline results.

#### 4.2.5 Reset the DML model

To mitigate concerns that model-specification choices within the DML framework could influence the baseline findings, we conduct robustness checks from multiple angles. First, we vary the functional form: whereas the baseline estimates rely on a partially linear DML specification, we additionally implement an interactive DML specification to assess the stability of the results. To reduce the disproportionate influence of extreme propensity-score weights in the interactive setting, we apply propensity-score trimming near the boundaries, using a trimming threshold of 0.01. Second, we vary the sample-splitting ratio, changing it from 1:4 in the baseline to 1:2 and 1:7 to examine whether alternative partition schemes affect inference. Third, we replace the machine-learning learner used for nuisance-function estimation, substituting random forests with LASSO regression and gradient boosting to test the sensitivity of the estimates to algorithm choice. Columns (1)–(5) of Table 6 report the results under these alternative specifications. Overall, re-specifying the DML framework does not alter the core conclusion that ZWCP significantly improves CCA, providing further evidence of robustness.

**Table 6:** Robustness check II.

Var	IRM	Kfolds = 3	Kfolds = 8	Lassocv	Gradboost	IV
	(1)	(2)	(3)	(4)	(5)	(6)
*zwcp*	0.048^***^	0.029^***^	0.024^***^	0.014^***^	0.029^***^	0.010^*^
	(0.001)	(0.004)	(0.004)	(0.003)	(0.004)	(0.054)
Control (single term)	Y	Y	Y	Y	Y	Y
Control (quadratic term)	Y	Y	Y	Y	Y	Y
City FE	Y	Y	Y	Y	Y	Y
Year FE	Y	Y	Y	Y	Y	Y
*N*	3,432	3,432	3,432	3,432	3,432	3,432

The dependent variable is the synergistic control of CO₂ and air pollutant emissions. Column (1) uses an interaction model. Columns (2) and (3) adjust the sample split ratio. Columns (4) and (5) use Lasso regression and gradient boosting, respectively, for prediction. Column (6) employs the instrumental variables method. Robust standard errors are reported in parentheses. *p < 0.01, *p < 0.05, *p < 0.1.

#### 4.2.6 Instrumental variable

The selection of ZWCP pilot areas is potentially non-random, which may introduce endogeneity concerns. We construct an instrumental variable as the interaction between regional average slope and river density, and use its natural logarithm as the instrument for ZWCP. With respect to relevance, this interaction is plausibly related to policy implementation intensity and likelihood through the joint influence of terrain and hydrological conditions: steeper terrain increases the difficulty and cost of waste collection, transport, and treatment—raising requirements for equipment and infrastructure investment and potentially affecting implementation efficiency—whereas higher river density elevates the risk of solid-waste pollution and the associated governance pressure, thereby strengthening local governments’ incentives to advance ZWCP. Regarding the exclusion restriction, both average slope and river density are natural geographic attributes that should not directly determine CCA; hence, their interaction (in logs) can plausibly satisfy the relevance and exogeneity conditions required for instrumental-variable identification. Column (6) of Table 6 shows that the estimated coefficient on the policy variable remains significantly positive and consistent with the baseline results, suggesting that the main finding is robust to addressing potential endogeneity.

## 5 Channel analysis

### 5.1 Source reduction

Following Li et al. (25), we construct a proxy for public environmental concern by summing the Baidu Search Index for the keywords “smog” and “environmental pollution” for each prefecture-level city. This indicator captures residents’ awareness of, and engagement with, environmental issues, which can facilitate source reduction on the consumption side by promoting greener consumption choices and low-carbon mobility, thereby reducing municipal waste generation and associated emissions (26). Moreover, heightened public concern may intensify social scrutiny and reputational pressure, reinforcing governmental regulation and corporate environmental compliance and generating complementarities between the consumption side and the governance side (27). Column (1) of Table 7 shows that ZWCP has a significantly positive effect on public environmental concern at the 1% level, suggesting that the policy increases residents’ attention to ecological issues and participation in environmental governance. By strengthening green awareness and behavioral constraints, ZWCP can thus encourage the diffusion of green consumption and low-carbon lifestyles, contributing to improvements in CCA.

**Table 7:** Channel analysis.

Var	Source reduction	Resource recycling	High-standard harmless treatment
	(1)	(2)	(3)
*zwcp*	0.065^**^	0.016^**^	0.010^**^
	(0.029)	(0.008)	(0.004)
Control (single term)	Y	Y	Y
Control (quadratic term)	Y	Y	Y
City FE	Y	Y	Y
Year FE	Y	Y	Y
N	3,432	3,432	3,432

The dependent variable is the synergistic control of CO₂ and air pollutant emissions. Robust standard errors are reported in parentheses. *p < 0.01, *p < 0.05, *p < 0.1.

### 5.2 Resource recycling

We measure the industrial circular-economy performance of each city using the industrial solid-waste comprehensive utilization rate, defined as the ratio of utilized industrial solid waste to total industrial solid waste generated. This measure directly reflects the extent of waste-to-resource conversion and cascading energy use within the urban industrial system and is widely used to gauge circular-economy outcomes. An increase in this utilization rate indicates that a larger share of waste is converted into recycled resources or substitute inputs, which can reduce primary resource extraction and energy-intensive production, thereby lowering both conventional pollutant emissions and carbon-emission intensity. Column (2) of Table 7 reports a significantly positive coefficient on ZWCP at the 1% level, indicating that the policy effectively promotes resource recycling and the synergistic utilization of industrial by-products. This evidence supports the view that ZWCP advances CCA by strengthening cross-industry circular linkages and cascading energy utilization.

### 5.3 High-standard harmless treatment

We use the harmless treatment rate of municipal solid waste as the key indicator for the high-standard harmless treatment channel. The indicator is defined as the share of municipal solid waste that receives harmless treatment in total municipal solid waste generated during the same period, capturing the quality of urban governance in collection, transport, treatment, and resource recovery. A higher harmless treatment rate reflects the establishment of a more intensive and integrated disposal network within a multi-waste coordinated governance system, which can enable simultaneous reductions in pollutants and carbon emissions at the end-of-pipe stage. Column (3) of Table 7 shows that ZWCP exerts a significantly positive effect on this indicator at the 1% level, implying that the policy improves the harmless treatment capacity and performance of municipal solid waste management. Through this pathway, ZWCP contributes to enhanced CCA within the urban governance system.

## 6 Heterogeneity test

To further explore the differential impacts of ZWCP, we conduct heterogeneity analysis based on regional location, city size, and resource endowment. The rationale for these classifications is grounded in both theoretical and institutional considerations. First, regional heterogeneity reflects systematic differences in economic development stages, environmental governance capacity, and policy implementation intensity across eastern, central, and western China. Second, city size is closely associated with administrative complexity and governance efficiency, which may affect policy effectiveness. Third, resource-based and non-resource-based cities differ significantly in industrial structure, path dependence, and transition costs, which are key factors influencing environmental policy outcomes.

### 6.1 Regional heterogeneity

Given China’s vast territory and pronounced regional disparities, cities differ substantially in resource endowments, stages of economic development, industrial structure, and environmental governance capacity. Consequently, the implementation and effectiveness of ZWCP may vary across regions. To examine regional heterogeneity, we follow the National Bureau of Statistics’ regional classification and partition the sample into two groups: the eastern region and the central–western region. We then estimate the model separately for each subsample to identify regional differences in the effect of ZWCP on CCA. As reported in Columns (1) and (2) of Table 8, the estimated policy effect is statistically significant in the central–western region but not in the eastern region. Relative to eastern cities, central and western cities typically exhibit a higher share of resource-intensive industries and larger legacy stocks of solid waste, implying greater scope for improvement after policy implementation. The results therefore suggest that ZWCP can exert stronger incentives in areas facing tighter resource constraints and greater ecological pressures. By contrast, many eastern cities already have comparatively advanced environmental governance and more mature solid-waste management systems, leaving more limited room for marginal improvement; accordingly, the estimated effect is not statistically distinguishable from zero. Overall, these findings indicate that the effectiveness of ZWCP is contingent on development stage and resource endowments, and its stronger performance in the central–western region highlights a role in “addressing weaknesses” and improving governance efficiency.

**Table 8:** Heterogeneity test results.

Var	Eastern	Central/western	Large	Small/medium	Resource	Non-resource
	(1)	(2)	(3)	(4)	(5)	(6)
*zwcp*	0.027	0.023^***^	0.031	0.022^***^	0.043	0.021^***^
	(0.108)	(0.004)	(0.073)	(0.005)	(0.085)	(0.003)
Control (single term)	Y	Y	Y	Y	Y	Y
Control (quadratic term)	Y	Y	Y	Y	Y	Y
City FE	Y	Y	Y	Y	Y	Y
Year FE	Y	Y	Y	Y	Y	Y
*N*	1,404	2028	1,188	2,244	1,380	2052

The dependent variable is the synergistic control of CO₂ and air pollutant emissions. Robust standard errors are reported in parentheses. *p < 0.01, *p < 0.05, *p < 0.1.

### 6.2 Size heterogeneity

City size not only shapes the distribution of economic and technological resources but also affects administrative constraints and the marginal effectiveness of policy implementation. Compared with large cities, small and medium-sized cities often face more pressing environmental and resource constraints in advancing ZWCP-related initiatives, which may translate into more targeted and forceful policy responses. Using total population as the measure of city size, we divide the sample into large cities and small/medium cities and estimate the model by subsample. Columns (3) and (4) of Table 8 show that ZWCP yields a more pronounced improvement in CCA in small and medium-sized cities, whereas the effect is not salient in large cities. One plausible explanation is that policy implementation in smaller cities may be more concentrated and flexible, enabling relatively rapid gains in solid-waste governance and circular utilization. In contrast, large cities typically feature more complex industrial structures and multi-layered governance arrangements, lengthening the policy transmission chain and potentially diluting synergistic gains, thereby weakening marginal effects.

### 6.3 Resource heterogeneity

Following the classification in the National Plan for Sustainable Development of Resource-Based Cities (2013–2020), we categorize the sample into resource-based and non-resource-based cities and conduct grouped regressions. The results, reported in Columns (5) and (6) of Table 8, indicate that ZWCP significantly improves CCA in non-resource-based cities, whereas the effect is not statistically significant in resource-based cities. This divergence likely reflects differences in economic structure and transition capacity. Non-resource-based cities are more often oriented toward services and high-technology industries, which tend to be less energy- and pollution-intensive; as a result, these cities may more readily leverage green transformation and institutional guidance to achieve concurrent reductions in conventional pollutants and carbon emissions. Moreover, their industrial structures and technology adoption patterns are typically more flexible, enabling faster responses to policy signals and amplifying policy impacts. By contrast, resource-based cities are frequently dependent on heavy industry and resource extraction, with energy systems dominated by fossil fuels. The scale of capital and technological investment required for structural upgrading is substantial, which may constrain the transmission of policy incentives and make it difficult to realize sizeable synergistic abatement effects in the short run. Lagged implementation capacity, weaker market responsiveness, and less-developed green innovation ecosystems may further attenuate the effectiveness of ZWCP in these cities.

## 7 Discussion

This study contributes to the literature in three aspects. First, it extends the research on coordinated environmental governance by providing the first causal evidence on the impact of the Zero-Waste City Pilot policy on synergistic control of carbon emissions and air pollution. Second, it enriches policy evaluation methods by introducing a double machine learning framework to address high-dimensional confounding and nonlinear relationships. Third, it contributes to the growing literature on environmental health by identifying synergistic emission reduction as an important intermediate mechanism linking environmental policy to public health benefits.

The findings also provide important insights into the mechanisms of integrated environmental governance. The evidence on source reduction, resource recycling, and harmless treatment suggests that ZWCP operates through a systemic approach rather than isolated interventions, highlighting the importance of coordinated policy design.

These results imply that promoting integrated policy frameworks that simultaneously target multiple environmental objectives can generate substantial co-benefits. In particular, strengthening public participation, improving circular economy systems, and facilitating structural transformation are key to enhancing policy effectiveness.

## 8 Conclusions and policy implications

### 8.1 Conclusion

The Zero-Waste City Pilot (ZWCP) serves as a critical policy instrument for advancing the dual-carbon agenda and promoting a circular-economy system, while also holding important implications for public health through the reduction of pollution-related environmental risks. Using panel data for 286 prefecture-level cities in China over the period 2011–2022, this study applies a double machine learning approach to identify the impact of ZWCP on the synergistic control of CO₂ and air pollutant emissions (CCA) and to examine heterogeneity in policy effects. The main findings are as follows. First, ZWCP significantly improves CCA in pilot cities, and the results remain robust across a battery of robustness checks. Second, mechanism tests indicate that the policy operates primarily through three channels—source reduction, resource recycling, and high-standard harmless treatment—thereby enhancing CCA. Third, heterogeneity analyses reveal that the synergistic gains are more pronounced in the central–western region, in small and medium-sized cities, and in non-resource-based cities.

### 8.2 Policy implications

#### 8.2.1 Strengthen coordinated governance for health

The evidence indicates that ZWCP, on average, significantly enhances city-level CCA, with important implications for reducing environmental exposure risks and improving public health. Policymakers should further refine the top-level design of ZWCP by explicitly embedding CCA-oriented objectives into the policy’s evaluation and accountability framework, thereby promoting a shift in solid-waste governance from a narrow focus on waste reduction toward a coordinated agenda of “pollution control–carbon mitigation–efficiency improvement.” Strengthening cross-departmental coordination is also essential: integrated governance arrangements should facilitate data sharing and policy alignment across relevant agencies (e.g., ecological environment, development and reform, industry and information technology, and housing and urban–rural development), enabling a full-process management system covering source reduction, circular utilization, and end-of-pipe treatment. Such coordinated governance can more effectively reduce pollutant emissions and population exposure to environmental hazards. In addition, a dynamic national mechanism for evaluating and disseminating ZWCP practices would help scale effective pilots and accelerate the transition from localized experimentation to broader implementation.

#### 8.2.2 Improve core mechanisms for risk reduction

Consistent with the mechanism evidence, policy design should prioritize the three key channels—source reduction, resource recycling, and high-standard harmless treatment—by strengthening the supporting institutional and technological infrastructure, thereby mitigating environmental exposure risks. On the production side, support for green manufacturing and cleaner production upgrades should be expanded, with greater emphasis on recyclability and reusability requirements at the product-design stage to promote green supply-chain coordination and reduce pollution at the source. On the recycling side, increased investment in R&D for solid-waste resource utilization technologies, together with the development of unified markets and trading mechanisms for recyclable resources, can facilitate closed-loop circulation from “waste–resource–product,” reducing reliance on primary resource extraction and associated emissions. On the treatment side, improving the harmless treatment rate of municipal solid waste remains crucial; local governments should be encouraged to adopt context-appropriate cleaner treatment technologies—such as waste-heat recovery from incineration and anaerobic digestion—to jointly advance pollution control, carbon mitigation, and environmental health protection.

#### 8.2.3 Promote targeted local implementation

The heterogeneity results suggest that ZWCP generates stronger synergistic effects in the central–western region, in small and medium-sized cities, and in non-resource-based cities, implying that the associated environmental and public health benefits may also vary across regions. These patterns underscore the importance of regionally differentiated policy packages. For central and western areas, stronger fiscal and policy-finance support could help expand waste-treatment infrastructure and circular-utilization capacity while fostering regional innovation networks, thereby reducing environmental exposure risks in relatively vulnerable areas. For small and medium-sized cities, strengthening implementation and regulatory capacity should be paired with streamlined and efficient governance models to improve policy responsiveness and enhance local environmental health outcomes. For resource-based cities, accelerating industrial restructuring and supporting green technological upgrading and resource regeneration industries are essential to reduce dependence on energy- and emissions-intensive sectors. Through tailored policy mixes aligned with local conditions, ZWCP can be implemented more sustainably, while also promoting more equitable distribution of environmental and health benefits across regions.

## Data Availability

The raw data supporting the conclusions of this article will be made available by the authors, without undue reservation.
